# Mesenchymal Stem Cell Therapy Using Human Umbilical Cord in a Rat Model of Autoimmune-Induced Premature Ovarian Failure

**DOI:** 10.1155/2020/3249495

**Published:** 2020-07-04

**Authors:** Zhe Wang, Quanwei Wei, Hao Wang, Linxiao Han, Hongjian Dai, Xiaoxin Qian, Hongliang Yu, Manqun Yin, Fangxiong Shi, Nianmin Qi

**Affiliations:** ^1^College of Animal Science and Technology, Nanjing Agricultural University, Nanjing 210095, China; ^2^Asia Stem Cell Therapies Co., Limited., Shanghai 201318, China; ^3^Changan Hospital of Dongguan, Dongguan 523000, China; ^4^Asia Stem Cell Regenerative Pharmaceutical Co., Ltd., Shanghai 201318, China

## Abstract

Premature ovarian failure (POF) is one of the principal causes of female infertility, and although its causes are complex and diverse, autoimmune deficiency may be involved. Human umbilical cord mesenchymal stem cells (UCMSCs) can be used for tissue regeneration and repair. Therefore, the present study was designed to determine the role of UCMSCs in immune factor-induced POF in rats. In this study, different concentrations of UCMSCs were injected into induced POF rats. Ovarian functions were examined by evaluating the estrus cycle, follicular morphology, hormonal secretion, and the proliferation and apoptosis of granulosa cells. Our results showed that the estrus cycle of rats returned to normal and follicular development was significantly improved after transplantation of UCMSCs. In addition, serum concentrations of 17-estradiol (E2), progesterone (P4), and anti-Müllerian hormone (AMH) increased significantly with treatment. Transplantation of UCMSCs also reduced the apoptosis of granulosa cells and promoted the proliferation of granulosa cells. All of these improvements were dose dependent. Furthermore, the results of related gene expression showed that transplanted human UCMSCs upregulated the expression of Bcl-2, AMH, and FSHR in the ovary of POF rats and downregulated the expression of caspase-3. These results further validated the potential mechanisms of promoting the release of cell growth factors and enhancing tissue regeneration and provide a theoretical basis for the clinical application of stem cells in the treatment of premature ovarian failure.

## 1. Introduction

As previously reported, many women suffer from premature ovarian failure (POF) before the age of forty, concomitant with amenorrhea, ovarian atrophy, low estrogen levels, and high levels of gonadotropins [1–4]. POF is caused by multiple factors, including heritage defects, autoimmunity, and environmental toxicity [5, 6]. Previous research has suggested that about 10% to 30% of POF disorders are caused by autoimmune mechanisms [7]. The pathologic characterizations of autoimmune ovarian disease (AOD) include inflammation, atrophy, and serum autoantibodies to ovarian antigens [8]. Therefore, a POF model has been established using autoimmune ovarian inflammation by injecting ovarian antigens into rats. The incidence of POF has shown an increasing trend in recent years, with younger women afflicted. Recently, the Women's Health Initiative (WHI) has revealed that the traditional treatment with hormone replacement therapy (HRT) could increase the incidence of breast cancer, endometrial cancer, cardiovascular disease, and stroke [9]. Therefore, it is of paramount importance to find a safer treatment for POF.

Stem cells have the potential to differentiate into various functional cells [10] and have been used in many clinical treatments for various diseases, including myocardial infarction [11], neurologic diseases [12], and diabetes [13]. Stem cells from different tissues—including bone marrow, amniotic fluid, and adipose tissue—also exert therapeutic effects on long-term infertility and ovarian damage [14–16]. Umbilical cord-derived mesenchymal stem cells (UCMSCs) have all the characteristics of common mesenchymal stem cells [17], are easy to obtain and culture in vitro, and have strong proliferative ability and low immunogenicity [18]. UCMSCs have an advantage over bone marrow and blood-derived mesenchymal stem cells in terms of material source and transport preservation [19, 20]. Studies have shown that they can successfully reach the ovary and play some functionally significant roles. Furthermore, their use can inhibit stromal cell apoptosis by secreting growth factors [21–23]. However, the exact protective roles of UCMSCs on damaged tissues remain unclear.

In the current study, we established a rat model of POF by injecting ovarian antigens into the rat subcutaneously, and via tail vein transplantation of UCMSCs, we confirmed their use as a cell therapy tool in the treatment of POF, and we demonstrated that the therapeutic effect was commensurate with increasing UCMSC concentrations. In this study, we preliminarily explored the possible mechanism(s) of UCSSCs to improve ovarian function, and our results provide a theoretical basis for the clinical application of stem cells in the treatment of POF.

## 2. Materials and Methods

### 2.1. Animals

One hundred and twenty female specific-pathogen-free- (SPF-) grade Sprague-Dawley (SD) rats at 8 weeks of age were used in this study after being purchased from the Qinglongshan Animal Breeding Farm (Nanjing, China). All procedures for animal handling were conducted under protocols approved by the Animal Welfare Committee of Nanjing Agricultural University.

### 2.2. Isolation and Culture of UCMSCs

The UCMSCs preparation (aStem-M-POF™) and related materials and samples were provided by Asia Stem Cell Regenerative Pharmaceutical Co., Ltd. After storage in liquid nitrogen, we thawed the UCMSCs in a 37°C water bath and rapidly centrifuged them at 1000 rpm/min for 5 min and then transferred the cells to a Petri dish. The *α*-MEM basal medium containing 10% fetal calf sera was replaced, the cells were cultured for 12 h and replaced, and the unattached cells were removed. Identification of mesenchymal stem cells was marked by flow cytometry for CD14, CD19, CD34, CD45, CD73, CD90, and CD105. In vitro induction of differentiation of mesenchymal stem cells included osteogenic differentiation, adipogenic differentiation, and chondrogenic differentiation. After identification, we changed the liquid every two days and observed the morphology and growth of the cells under an inverted microscope. When the cell fusion degree was 80%–90%, the mass was digested with TrypLE at 37°C for 5 min, and the morphology of the cells was observed under a microscope. When the cells were rounded and about to leave the bottle wall, we immediately terminated the digestion with an equal amount of medium, and the pipe was gently and repeatedly blown such that the cells were completely detached; a single cell suspension was then prepared and counted. We centrifuged the cells at 1000 rpm/min for 5 min, removed the supernatant, and added new PBS to adjust the cells to the desired concentration.

### 2.3. Ovarian Antigen

The whole ovarian tissues of rats were collected, weighed, rinsed with physiologic saline, and shredded and ground into a homogenate in a Tris-HCl buffer. In order to release ovarian proteins from the ovarian tissue, we performed ultrasonic pulverization with an amplitude of 14 microns for 5 min at low temperature (on ice) and then centrifuged the homogenate at low temperature (4000 rpm/min, 15 min; 10000 rpm/min, 5 min). The supernatant was removed, and the concentration was adjusted so that the ovarian tissue contained 20 mg per 0.1 mL of liquid. We used whole ovarian protein as the antigen for future use and mixed Freund's adjuvant when using it [24].

### 2.4. The POF Rat Model

To establish the autoimmune-induced POF model in rats, 120 healthy female rats 8 weeks of age were randomly divided into the control (*n* = 30) and model groups (*n* = 90). Rats in the model group were immunized by subcutaneous injection of 0.35 mL of ovarian antigen 3 times, once every 10 days. In the first immunization, an equal amount of Freund's complete adjuvant was added to the supernatant of the centrifuged ovarian tissue, and an equal amount of Freund's incomplete adjuvant was added to the supernatant of the centrifuged ovarian tissue for the second and third immunizations [25, 26].

### 2.5. Stem Cell Transplantation

Two weeks after ovarian antigen injection, rats exhibiting normal estrus cycles and stable hormone levels were selected as the blank control group with no processing. We then randomly divided 60 rats from the model group into 4 groups (groups A, B, C, and D) for tail vein injections, with 15 rats in each group. Group A was the vehicle control group (POF+NS), receiving a tail vein injection of 1 mL of PBS. Groups B (POF+low dose), C (POF+medium dose), and D (POF+high dose) were injected with UCMSCs at cell concentrations of 0.25 × 10^6^/mL, 1.00 × 10^6^/mL, or 4.00 × 10^6^/mL into the tail vein, respectively [27].

### 2.6. Examination of Estrus Cycles

The normal estrus cycle of rats consists of the following 4 consecutive stages: proestrus, estrus, metestrus, and diestrus. We determined the stages based upon the presence or absence of leukocytes, cornified epithelial cells, and nucleated epithelial cells. The vaginal cells were washed with saline and transferred to a glass slide, air dried, and stained with Giemsa dye solution. After the third immunization and treatment with UCMSCs, we observed the rat vaginal smears daily for 2 weeks, including at least 2 consecutive normal estrus cycles [28].

### 2.7. Ovarian Follicle Counting and Morphologic Analysis

Two weeks after modelling, to confirm whether our POF modelling was successful, we observed the morphological characteristics of ovarian follicles. 5 rats in the control group and the model group were randomly selected, and the animals were euthanized under ether anaesthesia. The ovaries we collected were fixed in 4% paraformaldehyde for 24 hours, dehydrated by a fractionated ethanol series, vitrified in xylene, embedded in paraffin, and embedded in paraffin. The paraffin blocks were serially cut at a thickness of 5 *μ*m per slice, and sections were stained with hematoxylin and eosin (H&E). After 3 weeks of treatment, the animals were euthanized under anaesthesia, the ovaries were collected and prepared for histologic examination, and we observed the sections under fluorescence microscopy. According to our previous study and references, we classified ovarian follicles as primordial, primary, secondary, early antral, and preovulatory [29–31].

### 2.8. Hormone Assay

To evaluate serum levels of E2, P4, and AMH, rats under anaesthesia were subjected to eyelid puncture, blood was collected after modelling and UCMSC transplantation, and blood samples were centrifuged at 3000 × g for 10 min. The serum was stored at −20°C for the measurement of biochemical indices. The serum levels of E2, P4, and AMH were determined with ELISA kits (Shanghai Xinfan Biological Technology Co., Ltd.) according to the instructions provided by the manufacturer. The sensitivities of the assays were 3 ng/L, 1 *μ*g/L, and 6 pg/mL for E2, P4, and AMH, respectively. The intra-assay and interassay coefficients of variation were less than 10% and 15%, respectively, for E2, P4, and AMH.

### 2.9. Immunohistochemical Staining of PCNA

We detected the proliferation of ovarian tissue cells by the cell proliferation PCNA immunohistochemistry test with the immunohistochemical SABC method. Paraffin sections were prepared by adhering tissue to microscope slides, and after dewaxing and hydration, we washed the sections 3 times with PBS for 5 minutes each. Antigen heat repair was in 10% trisodium citrate. After cooling to room temperature, the sections were again washed 3 times with PBS for 5 minutes each. We disposed of fresh 3% H_2_O_2_ with PBS and shook the sections for 30 minutes to remove the endogenous catalase. After shaking in PBS, normal goat serum blocking solution was added dropwise at room temperature for 1 hour. We removed excess liquid and added 1 antibody and incubated the sections overnight at 4°C. After shaking in PBS, biotinylated secondary antibody was added dropwise, and sections were incubated for 2 hours at room temperature. After shaking in PBS, we added the reagent SABC and incubated the sections for 1 hour at room temperature. After shaking in PBS, we allowed the color to develop using a DAB color developing kit. Finally, we counterstained with hematoxylin for 2 minutes and then differentiated with hydrochloric acid alcohol. We ultimately observed the sections with fluorescence microscopy.

### 2.10. Apoptosis Assay

We detected histochemically fragmented DNA by TUNEL using an in situ cell death assay kit. Fluorescein-labeled nucleotides were incorporated in situ into the 3′ end of the DNA strand breaks of apoptotic cells. According to the instructions, paraffin wax sections were dewaxed in xylene (twice, 5 minutes each time), and we gradually rehydrated the sections with ethanol (100%, 90%, 80%, and 75% consecutively). The slides were washed 3 times with PBS for 5 minutes each, and we incubated with proteinase K for 20 minutes at 37°C. Fifty *μ*L of TdT enzyme reaction mixture was added to the sample, and the entire coagulum was incubated in the dark for 30 minutes at 37°C in a humidified atmosphere. We washed the coagulum in PBS for 5 minutes, added streptavidin-fluorescein reagent to the sections, and incubated them in the dark for 1 hour at 37°C in a humidified atmosphere. After washing 3 times with PBS, the nuclei were stained with DAPI for 5 minutes, with the apoptotic cells in the ovary staining green [32]. We the observed the sections with fluorescence microscopy.

### 2.11. qRT-PCR

Total RNA in ovarian tissue was extracted using a TRIzol reagent for cDNA synthesis according to the AMH, Bcl-2, caspase-3, and FSHR gene sequences in GenBank. We designed the primers and probes (Table 1) using Premier 5.0 software, and the primers were synthesized by TSINGKE Biotechnology Co., Ltd. qRT-PCR amplification was performed using the AceQ® qPCR SYBR® Green Master Mix kit and an ABI 7500 real-time PCR system using common AMH, Bcl-2, caspase-3, and FSHR cDNA as templates. We used the GAPDH gene as an internal reference and the 2^-*ΔΔ*ct^ method to analyze the relative expression of genes.

### 2.12. Statistical Analyses

The data were analyzed using SPSS 23.0. The follicle numbers were expressed as mean ± standard deviation (SD) and were analyzed by Student's *t*-test and one-way ANOVA. Multigroup comparisons were analyzed using 1-way ANOVA. Independent data for 2 groups were analyzed by *t*-test, and the difference was statistically significant at *P* < 0.05.

## 3. Results

### 3.1. The Characterization of Human UCMSCs

**Figure 1:**
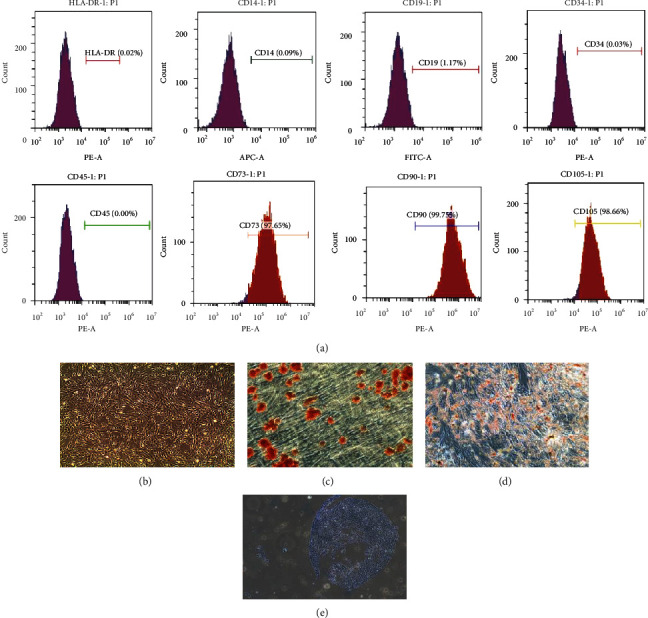
Cell surface markers and differentiation capability of UCMSCs. Cell surface markers were detected by FCM as described in Materials and Methods. (a) Flow cytometric analysis of human UCMSCs showing expression for CD14/CD19/CD34/CD45/CD73/CD90/CD105. (b) Newly revived UCMSCs. (c–e) Differentiated osteoblasts, adipocytes, and chondrocytes induced by UCMSCs.

The UCMSCs were small round cells that gradually became larger in culture then became fusiform, polygonal, and spindle-shaped. When cultured until the third day, the morphology of most cells became fusiform (Figure 1(b)). The molecular expression at the surface of the UCMSCs was detected using flow cytometry, and the results indicated a high expression of CD73/CD90/CD105 (>95%) and low expression levels of CD14/CD19/CD34/CD45/HLA-DR (<2%) (Figure 1(a)). We also demonstrated that UCMSCs still retained the surface marker characteristics of mesenchymal stem cells. Identification of the multidirectional differentiation capability of UCMSCs was made by in vitro induction, and these results showed that UCMSCs could differentiate into osteoblasts, adipocytes, and chondrocytes (Figures 1(c)–1(e)). This indicated that UCMSCs had the potential for multidirectional differentiation.

### 3.2. Ovarian Antigen Injection Causes Disorder in the Estrus Cycle of Rats

**Figure 2:**
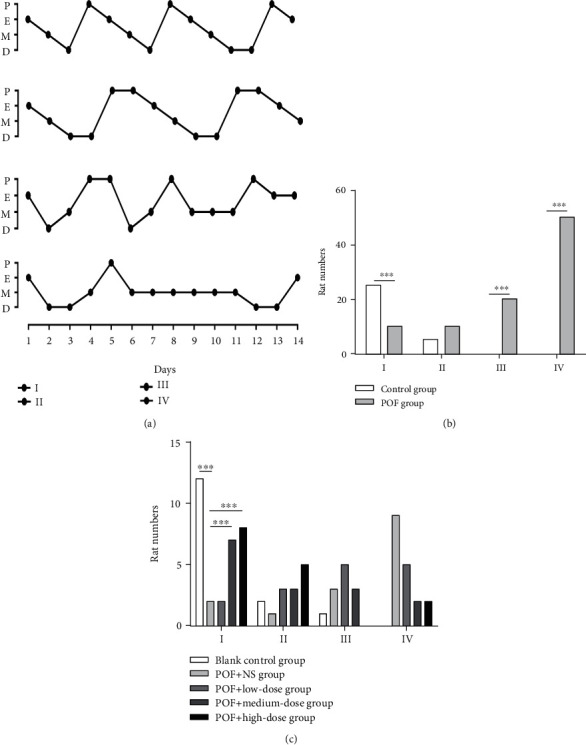
Effects of modeling and UCMSC transplantation on the estrus cycle of rats. (a) Four patterns of abnormal estrus cycles were graded with the severity of abnormality (I–IV) as follows: I: normal; II: regular cycles with a shortened estrus; III: irregular cycles with a prolonged diestrus and normal or prolonged estrus; IV: no cyclicity. The *y*-axis represents the cycle day in proestrus, estrus, metestrus, and diestrus. (b) The total numbers of rats from the control and model groups that were categorized into the various estrus patterns (I–IV), ^∗∗∗^*P* < 0.001. (c) The total numbers of rats from each group after UCMSC transplantation were categorized into the various estrus patterns (I–IV), ^∗∗∗^*P* < 0.001.

After ovarian antigen injection, the estrus cycles of rats in each group were examined daily for 2 weeks, and their patterns are shown in Figure 2(a). The degree of circulatory abnormality (I–IV) was classified as follows: I—normal; II—regular cycle with a shortened estrus; III—irregular cycle, persistent estrus, or prolonged estrus; and IV—no periodicity. In the control group, 83.3% of the rats had regular estrus cycles, whereas 87.5% of the rats in the modeling group experienced estrus cycle disorders, of which 63.3% had no regular estrus cyclicity (Figure 2(b)). These results showed that ovarian antigen injection caused disorders of the estrus cycle in rats.

### 3.3. Changes in Serum Hormone Levels after Ovarian Antigen Injection

**Figure 3:**
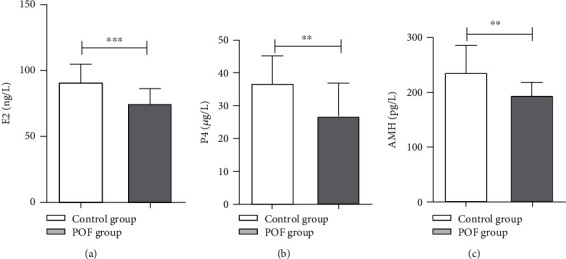
The levels of E2, P4, and AMH for each group (control group, POF group) after ovarian antigen injection. (a) Concentrations of E2 with ovarian antigen administration. (b) Concentrations of P4 with ovarian antigen administration. (c) Concentrations of AMH with ovarian antigen administration. ^∗∗^*P* < 0.01, ^∗∗∗^*P* < 0.001.

Two weeks after ovarian antigen injection, we obtained blood samples from the control group and one-third of the rats in the model group. Serum levels of E2 (*P* < 0.001), P4 (*P* = 0.0093), and AMH (*P* = 0.0017) were significantly lower in the model group relative to controls (Figures 3(a)–3(c), respectively). These results indicated that we established the POF animal model successfully.

### 3.4. Effects of Ovarian Antigen on Follicle Development

**Figure 4:**
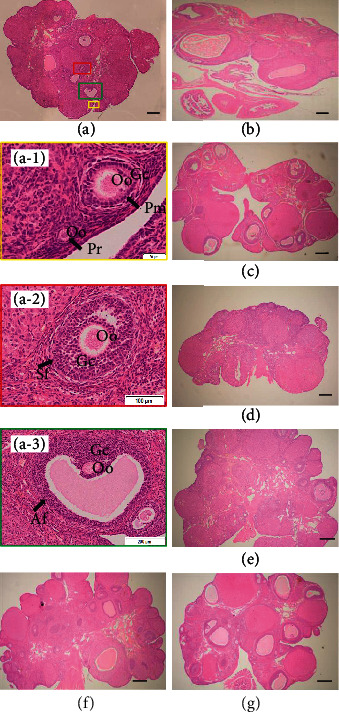
Ovarian histopathology using H&E staining. (a) Healthy rat ovarian. Photographs in differently colored individual square boxes marked in (a) were enlarged and marked with a-1, a-2, and a-3. Pr: primordial follicle; Pm: primary follicle; Sf: secondary follicle; Af: antral follicle; Oo: oocyte; Gc: granulosa cell. (b) Histopathologic examination of ovary tissues after ovarian antigen injection. (c) Rat ovary in the group of blank controls. (d) Rat ovary in the group of solvent controls. (e–g) Histopathologic examination of ovarian tissues after UCMSC transplantation. (e) Low-dose group. (f) Medium-dose group. (g) High-dose group. Black scale bar = 200 *μ*m.

The ovarian structure of the control group was intact and well developed. We observed follicles and corpora lutea at all levels. Clusters of primordial follicles can be observed in the superficial layer of the cortex; there are a large number of growing follicles in the deep layer of the cortex, surrounded by transparent bands and radial crowns around oocytes, and a gradually appearing follicle cavity, cumulus and follicle membrane, and large mature follicles can be observed near the surface of the ovary (Figure 4(a)). Observations of ovarian tissue morphology revealed that the POF group had decreased numbers of follicles at each stage of development; although clustered primitive follicles can also be observed, the number of growing and mature follicles is significantly reduced, granulosa cells are reduced, and the shape of the follicles is nonfull round and showed the atrophied ovaries (Figure 4(b)).

### 3.5. Transplantation of UCMSCs Improves the Estrus Cycle of POF Rats

After transplantation of UCMSCs, we examined the estrus cycles of the rats in each group within 2 weeks, and we noted that 93.3% of the blank control rats showed normal estrus cycles. In contrast, only 20.0% of the rats in the vehicle control group showed regular cycles after transplantation of UCMSCs (Figure 2(c)). In the low-dose group, 33.3% of the rats showed a regular circulation after transplantation of UCMSCs (Figure 2(c)), while in the medium-dose group, 66.6% of the rats showed regular circulation after transplantation (Figure 2(c)). Only 86.6% of the rats administered with the high dose showed regular cycles after transplantation (Figure 2(c)). Compared with the vehicle control group, the number of normal cycle rats in the medium-dose and high-dose groups was significantly increased (*P* < 0.001). Normal female rats have a regular estrus cycle that lasts 4 to 5 days, including the proestrus, estrus, metestrus, and diestrus [31]. In the second week of estrus cycle detection, it was found that the law of the estrus cycle was more and more obvious; that is, the law of the estrus cycle gradually recovered after transplantation. The results showed that the irregularity of the estrus cycles after UCMSC transplantation was improved significantly.

### 3.6. Histologic Examination of Ovarian Tissues following UCMSC Transplantation

We performed histologic examinations on all subgroups to assess ovarian tissue effects (Figures 4(c)–4(g)). The healthy ovary was observed to contain a large number of healthy follicles at all stages, including primordial follicles and primary follicles (Figure 4(a-1)), secondary follicles (Figure 4(a-2)), antral follicles (Figure 4(a-3)), and corpora lutea as shown in Figure 4. In contrast, the ovaries of the vehicle control group showed atrophied ovaries, and numbers of healthy follicles at each stage of development decreased while atretic follicles increased (Figure 4(d)). In contrast, the ovarian structure of rats after transplantation with UCMSCs was improved somewhat. The low-dose group had fewer follicles, partial atresia, corpora lutea, and fewer mature follicles (Figure 4(e)). We observed relatively intact ovarian structures in the middle-dose and high-dose groups that were well-developed, with visible follicles at all levels, and there were more corpora lutea and mature large follicles (Figures 4(f) and 4(g)). When we compared follicle counts with the statistics using the same follicle classification method, the results showed that the numbers of primordial follicles, primary follicles, mature follicles, and corpora lutea in the vehicle control group were significantly lower than those in the healthy control rats (*P* < 0.05), further validating the success of our model. The number of primordial follicles and primary follicles in the low-dose group was significantly reduced (*P* < 0.05), and compared with the vehicle control group, the numbers of primordial follicles, primary follicles, mature follicles, and corpora lutea were significantly increased in the high-dose group (*P* < 0.05). However, no significant differences were observed in the number of secondary follicles among the groups (Table 2).

### 3.7. Transplantation of UCMSCs Improves Hormone Secretion in POF Rats

**Figure 5:**
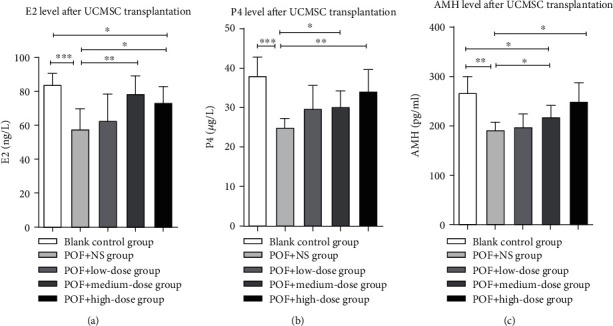
Serum concentrations of E2, P4, and AMH in each group during the 2 weeks after ovarian UCMSC transplantation. (a) E2 (estradiol), (b) P4 (progesterone), (c) AMH (anti-Müllerian hormone). ^∗^*P* < 0.05; ^∗∗^*P* < 0.01; ^∗∗∗^*P* < 0.001.

After 2 weeks of transplantation of UCMSCs, we obtained blood samples from rats in all groups and analyzed the effects of UCMSC transplantation on hormone secretion (E2, P4, and AMH) (Figures 5(a)–5(c)). Compared with the vehicle control group, serum levels of E2, P4, and AMH in the medium- and high-dose groups were significantly increased after UCMSC transplantation (*P* < 0.05). The hormone levels in the low-dose group were not significantly higher than those in the vehicle control group. Compared with healthy blank control rats, there were significant differences in hormone contents in the low-dose group (*P* < 0.01). Compared with the high-dose group, there was a significant difference in E2 content (*P* < 0.05), but there was no difference in P4 and AMH content. There were significant differences in P4 and AMH contents between the blank control group and the medium-dose group (*P* < 0.05). Our results demonstrated that the transplantation of UCMSCs promoted increases in E2, P4, and AMH and that the effect was dose dependent.

### 3.8. Transplantation of UCMSCs Promotes Ovarian Cellular Proliferation in POF Rats

**Figure 6:**
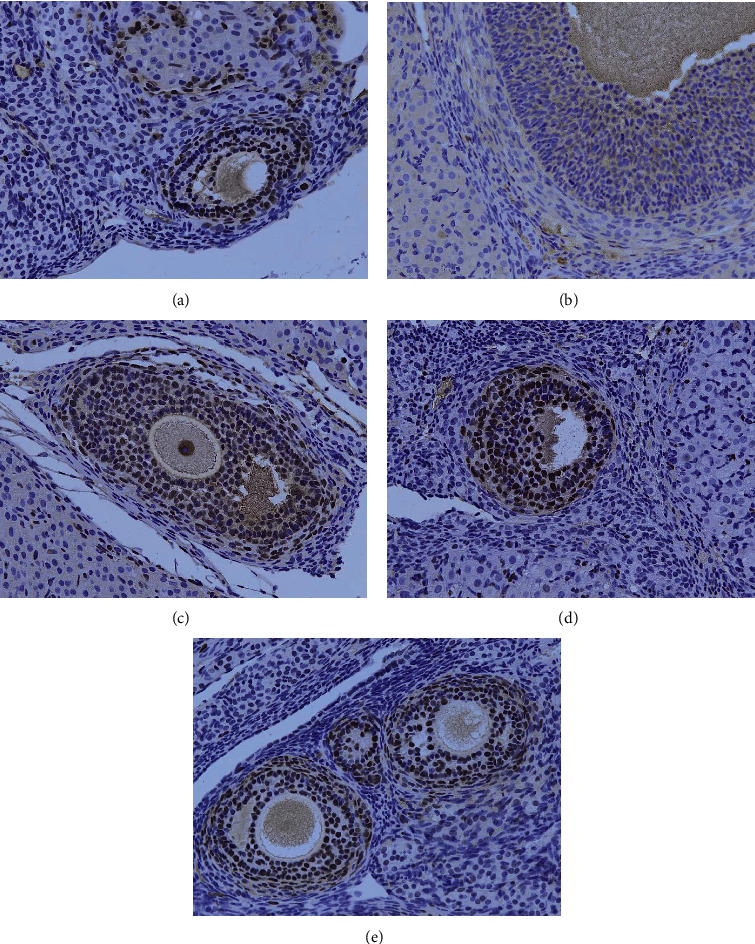
Cell proliferation in the ovaries as detected by PCNA. (a) Blank control group; (b) solvent control group; (c) low-dose group; (d) medium-dose group; (e) high-dose group.

Cell proliferation in ovarian tissue was detected by PCNA immunohistochemistry. After ovarian antigen injection, few proliferating cells were observed in the ovarian tissue of the vehicle control group (Figure 6(b)), while after transplantation of UCMSCs, the ovary of the healthy control group showed cell proliferation (Figure 6(a)). The cellular proliferation of the low-dose group was also less (Figure 6(c)); in contrast, the cell proliferation was significant in the ovarian sections of the medium-dose and high-dose groups (Figures 6(d) and 6(e)).

### 3.9. UCMSC Transplantation Reduced Ovarian Cell Apoptosis in POF Rats

**Figure 7:**
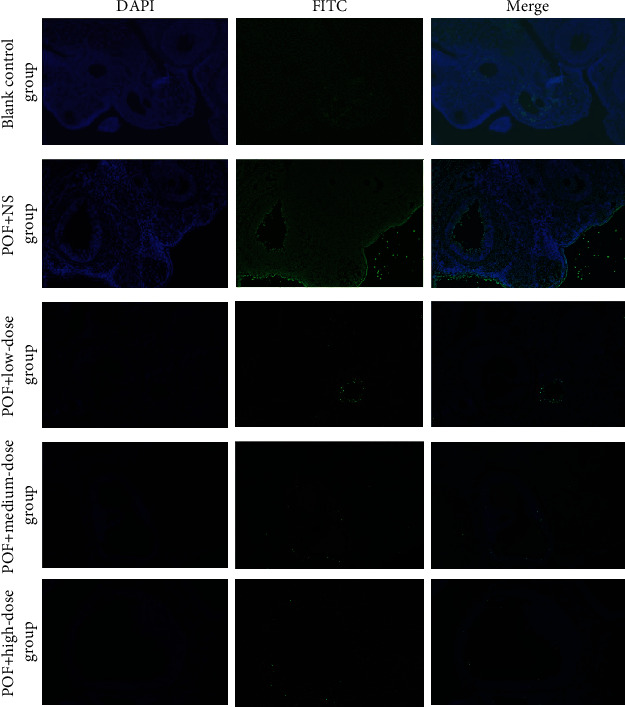
UCMSC transplantation reduced cellular apoptosis in ovaries of autoimmune-induced POF rats.

We observed cellular apoptosis in ovarian tissues by TUNEL assay. After ovarian antigen injection, a large number of apoptotic cells were observed in the ovarian tissue of the vehicle control group, the ovary of the healthy control group showed the healthiest follicles, and apoptosis did not increase significantly. According to our results, after 2 weeks of UCMSC transplantation, the number of apoptotic cells (FITC-positive cells) in the ovarian sections of the POF+medium-dose group and the POF+high-dose group was reduced compared with that of the vehicle control group (Figure 7).

### 3.10. UCMSCs Affect Follicular Development-Related Gene Expression

**Figure 8:**
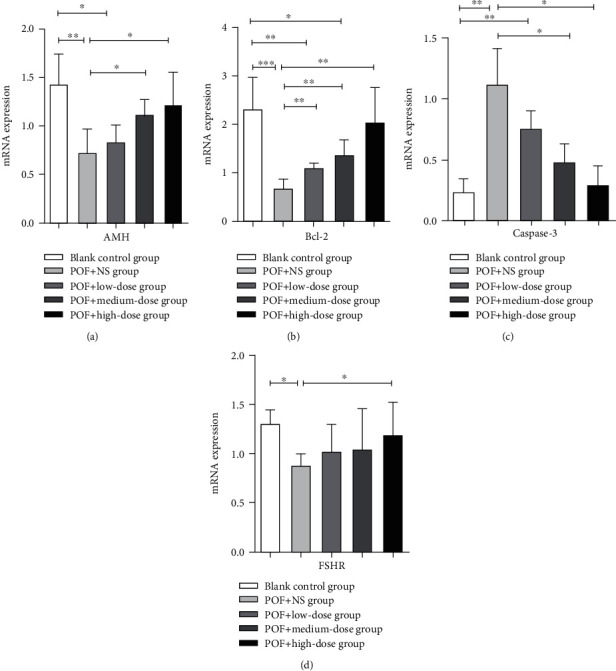
Expression of AMH, Bcl-2, caspase-3, and FSHR mRNA in rat ovaries. (a) AMH, (b) Bcl-2, (c) caspase-3, and (d) FSHR. ^∗^*P* < 0.05 and ^∗∗^*P* < 0.01.

The results of RT-PCR showed that there were significant differences in the expression levels of AMH, Bcl-2, caspase-3, and FSHR mRNA between the vehicle control group and the blank control group. Compared with the vehicle control group, the mRNA expression of Bcl-2 in all UCMSC transplantation groups was significantly increased (*P* < 0.05). Compared with the POF+NS group, the expression of AMH mRNA in the POF+medium-dose group and the POF+high-dose group was significantly increased, while the expression of caspase-3 mRNA was significantly decreased (*P* < 0.05), and FSHR mRNA expression was significantly increased in the high-dose group (*P* < 0.05) (Figure 8).

## 4. Discussion

Stem cells have great potential for repairing damaged tissues. With the application of tissue engineering in regenerative medicine and pluripotent cells, human embryonic stem cells (HuESCs) have become an important tool for the treatment of human degenerative diseases. However, HuESCs are severely limited in terms of ethics and safety in clinical applications. Human mesenchymal stem cells (HuMSCs) have also been reported to be useful in the treatment of irreversible genetic diseases [33]. To date, HuMSCs have had significant effects on the treatment of hereditary diseases in certain tissues and organs, including diabetes, spinal cord injury, and heart failure [11–13]. HuMSCs can avoid the ethical issues inherent in other modalities and reduce the likelihood of immune rejection—which has great prospects for the field of cell therapy.

In the treatment of POF, many types of stem cells have been shown to exert recovery effects, such as adipose stem cells (ADSCs) [34] and human endometrial stem cells (EnSCs) [19]. In the present study, human umbilical cord mesenchymal stem cells (HuCMSCs) were obtained and a single cell suspension was prepared then injected into the tail vein of the rats. HuCMSCs have been widely used in neurologic diseases and tissue regeneration. Moreover, there are no reports regarding tumor formation after treatment with UCMSCs. However, stem cells are commonly used to treat chemotherapy-induced POF. The therapeutic effects of stem cells on autoimmune injury-induced POF, however, remain unclear. Therefore, our aim was to clarify whether stem cells isolated from the human umbilical cord could restore autoimmune injury-induced ovarian dysfunction and explore the possible regulatory mechanism(s) of stem cells.

First, we used a subcutaneous injection of an ovarian antigen to cause damage to the rat ovary, thus establishing a POF model. The animal model after ovarian antigen treatment proved that ovarian function was significantly impaired as we determined estrus cycles and E2, AMH, and P4 hormone levels and examined the pathology of ovarian tissues. Our study showed that the levels of P4, AMH, and E2 in the serum of the modeled group were significantly lower than those of the control group, and the rats showed irregular estrus cycles after antigen injection. These results are consistent with POF symptoms. In addition, it has been reported that follicular development is affected by the endocrine system [31, 35]. These endocrine disorders may affect follicular reserve and follicular development, leading to ovarian pathologies such as POF. The ovarian tissues in the model group showed obvious atrophy, along with the decreased sinusoid follicles, and an increase in the number of atretic follicles. The atrophic ovary is mainly composed of interstitial cells in the fibrous matrix, with a reduced number of follicles at each developmental stage, follicular luteinization, reduced granulosa cells, and nonfull round shapes. Collectively, ovarian antigen injection had a negative impact on ovarian function, and we demonstrated that the POF model was successfully established by using this method.

After 2 weeks of UCMSC transplantation, ovarian function of POF rats was improved, including the estrus cycle tending to become more regular. Hormone levels such as E2, AMH, and P4 in the blood were significantly elevated. These data indicated that the improved ovarian endocrine function was possibly affected by UCMSC transplantation. However, our data showed that the medium-dose group exhibited the highest E2 levels but not significantly different from the high-dose group. In addition, observation of ovarian histopathology revealed an increase in the number of healthy follicles and a decrease in GC apoptosis in ovarian tissue of sinusoidal follicles. It has been reported that ovarian GCs play a key role in oocyte production [36], and our GC apoptosis results showed that UCMSC transplantation reduced GC apoptosis in large secondary and antral follicles. The inhibitory effect of transplanted UCMSCs on the apoptosis of GCs may promote the recovery of follicles in POF rats. In addition, we found that the effects of UCMSCs on POF rats were dose dependent. As we increased the UCMSC dose, estrous cycle characteristics of POF rats became more regular, and serum estrogen content also returned to the blank control group levels. Histopathologic observations of the ovary clearly showed that the follicular development of the medium-dose group and the high-dose group was better than that of the low-dose group, and we observed corpora lutea and mature large follicle formation. In conclusion, UCMSC transplantation showed dose-dependent effects on improving ovarian follicular development in POF rats.

Many previous studies have shown that stem cells can differentiate into specific tissues or organ cells and that stem cells can be transplanted into a specific microenvironment stimulated by ecology and released by cell growth factors to promote regeneration of surrounding tissues [37, 38]. Previous studies have shown that HuMenSC can be induced to differentiate into ovarian tissue-like cells under the niche stimulation of POF mice [14]. Hormones and ovarian cytokines, growth factors, and intracellular proteins can all regulate follicle growth [39]. All these factors may contribute to the recovery of ovarian function mediated by UCMSC transplantation in our experiments, but this needs further research in the future. In our present study, we found that follicular development was improved after UCMSC transplantation. Cellular proliferation was also improved, especially in developing follicles. The differentiation of UCMSC may not be the direct effect to improve the ovarian function. However, it may be possible by improving the internal environment of the ovaries [40, 41]. Bcl-2 and caspase-3 are 2 key factors in the regulation of apoptosis. Bcl-2 is an important apoptosis-inhibiting gene and plays a regulatory role through its encoded protein. When the Bcl-2 protein binds to a homodimer, it can inhibit apoptosis caused by various stimuli and damage [42]. The caspase family also plays an important role in mediating apoptosis [43]. Caspase increases the activity of the Ca^2+^/Mg^2+^-dependent endonuclease, which is affected by the negative regulation of PARP, and cleaves DNA between nucleosomes, causing apoptosis [44]. Bax and caspase-3 are required for ovarian follicle development and atresia [30]. Our results showed that after transplantation of UCMSCs, Bcl-2 was negatively correlated with caspase-3. Our data showed that transplantation of UCMSCs upregulated ovarian Bcl-2 and downregulated caspase-3 mRNA expression. We also found that the increase in the number of primordial follicles and primary follicles might be influenced by the relevant growth factors. It has been reported that follicle-stimulating hormone receptor reduction or dysfunction on the follicular cell membrane is not sensitive to FSH stimulation, leading to follicle cessation of growth and POF [45]. Regulation of follicular development depends on the reproductive endocrine axis and local ovarian regulatory factors. AMH, one of the regulatory cellular factors in the ovary, plays a very important role in the growth of follicles and can inhibit the initial recruitment of follicles and reduce the sensitivity of follicles to FSH by inhibiting the activity of aromatase. In the present study, the mRNA expression of AMH and FSHR was significantly decreased after ovarian antigen injection. The expression of AMH was significantly upregulated by UCMSCs, and FSHR was upregulated only in the high-dose group. Collectively, transplantation of human UCMSCs upregulated the expression of Bcl-2, AMH, and FSHR in the ovary of POF rats and downregulated the expression of caspase-3. These results further validate the underlying mechanisms that promote the release of cellular growth factors and thereby promote tissue regeneration. However, the elucidation of the recovery of ovarian function after transplantation of UCMSCs and the exact mechanism of regulation of related cells and cytokines still require further future research.

## 5. Conclusions

In summary, transplantation of human UCMSCs improved the ovarian dysfunction caused by autoimmunity in POF rats. UCMSC transplantation regulated the estrus cycle of POF rats and improved their endocrine function. It also inhibited apoptosis of ovarian cells, promoted follicular development, and regulated the expression of related genes. The specific mechanism underlying the effects of human UCMSC in the treatment of POF remains to be elucidated.

## Figures and Tables

**Table 1:** Sequences of PCR primers and length of products.

Gene	Sequence of primer (5′-3′)	Length of product
Bcl-2	Sense: AGCCTGAGAGCAACCGAACG	125 bp
	Antisense: AGCCTGAGAGCAACCGAACG	
Caspase-3	Sense: AACTGGACTGTGGCATTGAGA	187 bp
	Antisense: AGGTGGAGTCATAGGAAAAGGAC	
AMH	Sense: GGGAGCAAGCCCTGTTAGTG	246 bp
	Antisense: AGCGGGAATCAGAGCCAAA	
FSHR	Sense: AAGCCCAGATTTACAGGACAG	113 bp
	Antisense: AAGAGGGACAAGCACGTAACTA	
GAPDH	Sense: ACGGCAAGTTCAACGGCAC	145 bp
	Antisense: ACGCCAGTAGACTCCACGACAT	

**Table 2:** Follicular and luteal counts from ovaries of rats with or without UCMSC transplantation.

Group	Primordial follicles	Primary follicles	Secondary follicles	Mature follicles	Corpus luteum
Blank control	598.3 ± 36.91	95.25 ± 10.99	53.75 ± 7.16	6.750 ± 0.48	13.75 ± 1.11
Group A	428.3 ± 39.54^a^	47.75 ± 5.98^a^	42.00 ± 3.19	2.857 ± 0.51^a^	10.71 ± 0.57^a^
Group B	456.3 ± 34.37^a^	53.00 ± 8.52^a^	44.50 ± 3.60	4.333 ± 0.80	10.50 ± 0.99
Group C	493.8 ± 33.51	58.50 ± 6.61^a^	42.00 ± 4.14	5.000 ± 0.52^ab^	11.67 ± 1.20
Group D	559.7 ± 23.90^b^	71.60 ± 6.35^b^	49.33 ± 1.71	5.833 ± 1.01^b^	14.50 ± 1.46^b^

^\a^ *P* < 0.05, compared with healthy mice; ^b^*P* < 0.05, compared with the vehicle control group (group A). Data are presented as means + SD. (group: A, POF+NS group; B, POF+low-dose group; C, POF+medium-dose group; and D, POF+high-dose group).

## Data Availability

The data used to support the findings of this study are included within the article.
